# Distinct diversity of skin cell populations of rhinophyma and hypertrophic scar illustrated by scRNA-seq

**DOI:** 10.3389/fimmu.2026.1703469

**Published:** 2026-03-12

**Authors:** Qiannan Li, Lanxin Geng, Xufeng He, Xiangyu Wei, Cheng Zhang, Yiyang Hu, Xiang He, Huimin Zhang, Wuqing Wang

**Affiliations:** 1 Department of Dermatology, Shuguang Hospital Affiliated to Shanghai University of Traditional Chinese Medicine, Shanghai, China; 2 Department of Acupuncture, Shuguang Hospital Affiliated to Shanghai University of Traditional Chinese Medicine, Shanghai, China; 3 Key Laboratory of Liver and Kidney Diseases (Ministry of Education), Shanghai Key Laboratory of Traditional Chinese Clinical Medicine, Shanghai, China; 4 Institute of Liver Diseases, Shuguang Hospital affiliated to Shanghai University of Traditional Chinese Medicine, Shanghai, China

**Keywords:** endothelial cells, fibrosis, hypertrophic scar, rhinophyma, scRNA-seq

## Abstract

**Introduction:**

The clinical manifestations and presentation of rhinophyma closely resemble those of hypertrophic scar tissue, both presenting as firm, fibrotic growths. Despite this phenotypic similarity, a critical divergence is observed following surgical intervention: the affected skin in rhinophyma can revert to its normal state without scar recurrence, a favorable outcome starkly contrasting with the behavior of hypertrophic scars. The underlying mechanisms for this phenomenon have yet to be elucidated. The aim of this study is to uncover the cellular and molecular disparities between these two pathological conditions using single-cell sequencing technology to resolve this clinical paradox. The objective of this study is to compare the single-cell transcriptomic profiles of rhinophyma and hypertrophic scar tissues to identify key cell types and molecular pathways that may account for the distinct healing fate of rhinophyma post-surgery and provide novel insights for the prevention and treatment of hypertrophic scars.

**Methods and results:**

We isolated single cells from rhinophyma and hypertrophic scar tissues and conducted transcriptomic analysis using high-throughput single-cell RNA sequencing technology. Employing bioinformatics methods, we analyzed the data to identify differentially expressed genes and cellular subpopulations. Our results indicate that epithelial cells in hypertrophic scar tissues exhibit distinct fibrotic characteristics not observed in rhinophyma tissues. Vascular structures in hypertrophic scar tissues are enveloped by a significant number of stromal cells, in contrast to the leaky vascular profiles observed in rhinophyma tissues. Furthermore, keratinocytes in hypertrophic scars display disordered proliferation, while the number of immune cells in rhinophyma is significantly higher than in hypertrophic scar tissues.

**Conclusion:**

These findings reveal fundamental differences in cellular composition and functionality between rhinophyma and hypertrophic scar tissues, the intrinsic differences in the tissue microenvironment predispose them to divergent healing trajectories following the same insult, offering new perspectives on the healing process of rhinophyma post-surgery and potentially providing molecular targets for the development of scar prevention strategies.

## 1 Introduction

Hypertrophic scar and rhinophyma are two common skin fibrosis diseases, the formation mechanisms of which are related to the abnormal deposition of connective tissue (1). Hypertrophic scar is an abnormal wound healing process caused by external injury, characterized by fibrotic proliferative inflammatory reactions due to excessive collagen deposition (2). Rhinophyma, on the other hand, is a skin fibrosis caused by chronic inflammation, affected by neurovascular and immune dysregulation (3). Despite their distinct etiologies, the clinical manifestations and presentation of these two conditions closely resemble each other. Both can present as firm, raised, erythematous, and rigid tissue masses on the skin surface, sharing key phenotypic features of hyperplasia and fibrosis. However, a critical divergence is observed in their response to surgical intervention: although both involve chronic inflammation and fibrosis, rhinophyma typically heals normally after surgery, whereas hypertrophic scar exhibits an abnormal healing process and a high risk of recurrence (4). This paradoxical observation—phenotypic similarity but divergent healing outcomes—forms the central scientific question of this study. The underlying mechanisms that account for this fundamental difference remain largely unknown.

To understand this paradox, it is essential to consider the well-studied mechanisms of hypertrophic scar formation, which is influenced by various factors, such as wound tension, genetic factors, hormone levels, and lifestyle. Multiple hypotheses have been proposed, including the dysregulation of inflammatory signaling pathways, TGF-β/Smad signaling pathways, and YAP/TAZ signaling pathways (2). In addition, the high infiltration rate of profibrotic immune cells (such as M2 macrophages and Th2 cells) is associated with the formation of hypertrophic scars, and these cells promote the activation of fibroblasts through the TGF-β1 signaling pathway. The increased expression of pro-inflammatory cytokines IL-6 and IL-8, and the decreased expression of anti-inflammatory cytokine IL-10, promote scar formation through JAK/STAT signaling pathways (1). IL-6 also promotes epithelial-mesenchymal transition (EMT) through the JAK/STAT pathway, further promoting the formation of hypertrophic scars. Studies have shown that keratinocytes in hypertrophic scars can mediate the role of fibroblasts in wound healing by secreting TGF-β, IL-1, and other factors, stimulating the proliferation of dermal fibroblasts or producing more extracellular matrix (ECM) signals (3). It is worth noting that endothelial dysfunction is associated with the formation of hypertrophic scars (4).

Regarding rhinophyma, studies have shown that various immune cells are activated in all its subtypes. Erythematotelangiectatic rhinophyma (ETR) is dominated by Th1/Th17 polarized immune cells, with significant upregulation of cytokines such as IFN-γ and IL-17, and an increase in macrophages and mast cells in all three Rhinophyma subtypes. Particularly, B cells increase in antibody production as they transition to rhinophymatous rosacea, indicating that Th1/Th17 polarization inflammation and macrophage and mast cell infiltration are significant markers of rhinophyma (5).

Given the marked clinical similarities yet divergent healing outcomes between rhinophyma and hypertrophic scar, we hypothesize that the impact of fibrotic inducers such as TGF-β in Rhinophyma on wound healing requires further investigation, rhinophyma can heal normally after surgery, while hypertrophic scar shows an abnormal healing process (6). This suggests that the impact of fibrotic inducers such as TGF-β in Rhinophyma on wound healing requires further investigation. By comparing rhinophyma and hypertrophic scar, we hope to explore the reasons for the normal healing after rhinophyma surgery, with the expectation that scars can also heal normally after surgery, or that intervention at the initial stage of trauma can prevent scar formation. To better understand the mechanisms of scar formation, we plan to conduct single-cell sequencing analysis of endothelial cells, smooth muscle cells, pericytes, fibroblasts, keratinocytes, and immune cells in normal skin, hypertrophic scar, and rhinophyma, to obtain the transcriptional profile of scars formed after trauma. We look forward to discovering new mechanisms through this study, providing new ideas and strategies for the prevention and treatment of scars.

## 2 Materials and methods

### 2.1 Clinical sample collection

This study was approved by the Ethics Committee of Shuguang Hospital Affiliated to Shanghai University of Traditional Chinese Medicine (No.2024-1488-071–01 and No.2024-1598-181-01). Written informed consent was obtained from all participants prior to sample collection. Human skin samples were surgically obtained under standard clinical procedures: hypertrophic scar tissue was collected during elective plastic surgery; rhinophyma specimens were harvested via five-blade scarification surgery using five-blade scalpel (inter-blade spacing: 0.8 mm; incision depth: 0.5-0.8 mm). All patients were histologically confirmed to have rhinophymatous rosacea through lesional biopsy, with pathology-confined nasal involvement; Normal skin was acquired from donor undergoing routine circumcision. The sampling sites for hypertrophic scar tissue in clinical patients were located in the central region of the lesion, with each sample measuring 0.5 cm3. A total of five samples were obtained. For patients diagnosed with rhinophyma, samples were taken from the rhinophyma area, each also measuring 0.5 cm3, with a total of five samples collected. In the case of healthy individuals, samples were obtained from normal skin, each measuring 0.25 cm3. Two samples were collected from each individual, resulting in a total of ten samples. All specimens were immediately processed for analysis following protocols compliant with the Declaration of Helsinki. Patients had received no prior topical/systemic treatments affecting the sampled areas for ≥4 weeks before surgery.

For the single-cell RNA sequencing (scRNA-seq) analysis, a subset of these samples was utilized, comprising one sample each from a single individual representing the three conditions: healthy skin (Skin-H), hypertrophic scar (HS-H), and rhinophyma (ROS). Thus, the scRNA-seq dataset is derived from three total samples (N = 1 per condition) and serves as an exploratory, hypothesis-generating investigation. Detailed demographic and clinical characteristics of all patients and samples are provided in **Supplementary Table 1**.

### 2.2 Preparation of single-cell suspensions

Single-cell RNA sequencing (scRNA-Seq) experiment was performed by experimental personnel in the laboratory of NovelBio Co.,Ltd. The tissues were surgically removed and kept in MACS Tissue Storage Solution (Miltenyi Biotec) until processing. The tissue samples were processed as described below. Briefly, samples were first washed with phosphate-buffered saline (PBS), minced into small pieces (approximately 1mm^3^) on ice and enzymatically digested with 0.75mg/mL collagenase I, 2mg/mL collagenase IV, 0.2mg/ml hyaluronidase and 75 U/mL DNase I (Worthington) for 45 min at 37 °C, with two rounds. After digestion, samples were sieved through a 70µm cell strainer, and centrifuged at 300g for 5 min. After washing with PBS containing 0.04% BSA, the cell pellets were re-suspended in PBS containing 0.04% BSA and re-filtered through a 35μm cell strainer. Dissociated single cells were then stained with AO/PI for viability assessment using Countstar Fluorescence Cell Analyzer.

### 2.3 Single-cell sequencing

The scRNA-Seq libraries were generated using the 10X Genomics Chromium Controller Instrument and Chromium Single Cell 3’ V3.1 Reagent Kits (10X Genomics, Pleasanton, CA). Briefly, cells were concentrated to approximately 1000 cells/uL and loaded into each channel to generate single-cell Gel Bead-In-Emulsions (GEMs). After the RT step, GEMs were broken and barcoded-cDNA was purified and amplified. The amplified barcoded-cDNA was fragmented, A-tailed, ligated with adaptors and index PCR amplified. The final libraries were quantified using the Qubit High Sensitivity DNA assay (Thermo Fisher Scientific) and the size distribution of the libraries were determined using a High Sensitivity DNA chip on a Bioanalyzer 2200. All libraries were sequenced by illumina sequencer (Illumina, San Diego, CA) on a 150 bp paired-end run.

### 2.4 Single-cell RNA statistical analysis

scRNA-seq data analysis was performed by NovelBio Co.,Ltd. with NovelBrain Cloud Analysis Platform. We applied with default parameter filtering the adaptor sequence and removed the low quality reads to achieve the clean data (7). Then the feature-barcode matrices were obtained by aligning reads to the human genome (GRCh38 Ensemble: v104) using CellRanger v6.1.1. We applied the down sample analysis among samples sequenced according to the mapped barcoded reads per cell of each sample and finally achieved the aggregated matrix. Cells contained over 200 expressed genes and mitochondria UMI rate below 20% passed the cell quality filtering and mitochondria genes were removed in the expression table. Seurat package (v4.0.3) was used for cell normalization and regression based on the expression table according to the UMI counts of each sample and percent of mitochondria rate to obtain the scaled data. PCA was constructed based on the scaled data with top 2000 high variable genes and top 10 principals were used for tSNE construction and UMAP construction. Utilizing graph-based cluster method, we acquired the unsupervised cell cluster result based the PCA top 10 principal and we calculated the marker genes by FindAllMarkers function with wilcox rank sum test algorithm under following criteria: 1. log2FC > 0.25; 2. *P* < 0.05; 3. min.pct > 0.1. In order to identify the cell type detailed, the clusters of same cell type were selected for re-tSNE analysis, graph-based clustering and marker analysis.

### 2.5 Pseudo-time trajectory analysis

We applied the Single-Cell Trajectories analysis utilizing Monocle2 (http://cole-trapnell-lab.github.io/monocle-release) using DDR-Tree and default parameter. Before Monocle analysis, we select marker genes of the Seurat clustering result and raw expression counts of the cell passed filtering. Based on the pseudo-time analysis, branch expression analysis modeling (BEAM Analysis) was applied for branch fate determined gene analysis.

### 2.6 Cell communication and SCENIC analysis

To enable a systematic analysis of cell–cell communication molecules, we applied cell communication analysis based on the Cell Phone DB (8), a public repository of ligands, receptors and their interactions. Membrane, secreted and peripheral proteins of the cluster of different time point was annotated. Significant mean and Cell Communication significance (p-value<0.05) was calculated based on the interaction and the normalized cell matrix achieved by Seurat Normalization. To assess transcription factor regulation strength, we applied the Single-cell regulatory network inference and clustering (pySCENIC, v0.9.5) workflow, using the 20-thousand motifs database for RcisTarget and GRNboost (9).

### 2.7 Gene enrichment and differential gene expression analysis

To characterize the relative activation of a given gene set such as pathway activation, we performed Gene Enrichment Analysis (QuSAGE, v2.16.1) analysis (10). To identify differentially expressed genes among samples, the function Find Markers with wilcox rank sum test algorithm was used under following criteria.

### 2.8 Go biological process and pathway analysis

Gene ontology analysis was performed to facilitate elucidating the biological implications of marker genes and differentially expressed genes (11). We downloaded the GO annotations from NCBI (http://www.ncbi.nlm.nih.gov/), UniProt (http://www.uniprot.org/) and the Gene Ontology (http://www.geneontology.org/). Fisher’s exact test was applied to identify the significant GO categories and FDR was used to correct the p-values. Pathway analysis was used to find out the significant pathway of the marker genes and differentially expressed genes according to KEGG database. We turn to the Fisher’s exact test to select the significant pathway, and the threshold of significance was defined by P-value and FDR (12).

### 2.9 ssGSEA analysis and CytoTRACE analysis

We applied the ssGSEA function of GSVA package based on the NEC geneset and AC geneset which was constructed by the differentially expressed gene of Adenocarcinoma and Paracancerous expression data (13). We applied CytoTRACE analysis for predicting differentiation state with default parameter (14). The original data have been uploaded to the public database Figshare (Macmillan, UK) and the corresponding access number, DOI: 10.6084/m9.figshare.30446972, has been provided.

### 2.10 Western blot

Protein extracts were isolated from human tissue samples (healthy skin, hypertrophic scar, and rhinophyma) using RIPA lysis buffer (Thermo Fisher Scientific) supplemented with protease and phosphatase inhibitors (Roche). Protein concentrations were quantified via BCA assay (Pierce). Equal amounts of protein (20–30 μg per lane) were separated by SDS-PAGE on 10–12% gels and transferred to PVDF membranes (Millipore). Membranes were blocked with 5% non-fat milk in TBST (Tris-buffered saline with 0.1% Tween-20) for 1 h at room temperature, followed by incubation with primary antibodies at 4 °C overnight. Primary antibodies included anti-COL1A1 (No.A22412), anti-SERPINE1 (No.A19096), anti-β-Actin (NoAS014), anti-F2R (NoA5641), anti-MDK (No.A0251) were purchased from Abconl, anti-ACKR1 (No.PH4105S), anti-THBS1 (No.TD6848S), anti-POSTN (No.T56911S), anti-DCN (No.TD6543S), anti-CXCL2 (No.PY88673S) were purchased from Abmart, and anti-CD3 (No.GB11014) were purchased from Service, as a loading control. After washing with TBST, membranes were incubated with HRP-conjugated secondary antibodies (1:5000, Jackson ImmunoResearch) for 1h at room temperature. Protein bands were visualized using enhanced chemiluminescence (ECL, Advansta) and imaged on a ChemiDoc system (Bio-Rad). Densitometric analysis was performed using Image Lab Software (Bio-Rad, v6.1).

### 2.11 Immunofluorescence

Immunofluorescence staining was performed on formalin-fixed paraffin-embedded human hypertrophic scar and normal scar biopsies. Tissue sections were deparaffinized and rehydrated followed by heat-induced antigen retrieval in citrate buffer at pH 6.0 for ~10 min. Antibodies were applied, including mouse anti-ADAM12 (sc293225, Santa Cruz) (1:50), rabbit anti-NREP (bs-0427R, Bioss) (1:100), or rabbit anti-SMA (ab124964, Abcam) (1:300) were incubated overnight at 4 °C. Sections were washed with PBS 3 times, and then labeled with Alexa Fluor 488 (A-11029, ThermoFisher) and 555 (A32732, ThermoFisher) labeled secondary antibodies (1:5000). Slides were coverslipped, using DAPI containing aqueous mounting medium. Images were obtained using a Nikon A1 + confocal laser-scanning microscope.

### 2.12 Statistical analysis

Statistical analyses were performed using GraphPad Prism (version 8). All data are presented as the mean ± SEM. We determined the data for normal distribution and similar variance between groups. The differences between 2 groups were compared by 2-tailed unpaired or paired Student’s t-test. For comparisons of more than 2 groups, one-way ANOVA with Tukey’s *post hoc* test or two-way ANOVA with a *post hoc* Holm–Sidak’s multiple comparisons test was performed. Two-sided permutation test without multiple comparison adjustments was used for GSEA analysis. When the data were not normally distributed or exhibited unequal variances between groups, we performed statistical analysis with two-tailed Mann–Whitney U test. A significance level of *P* < 0.05 was applied to determine notable differences unless otherwise specified.

## 3 Results

### 3.1 Single-cell analysis of skin conditions

**Figure 1:**
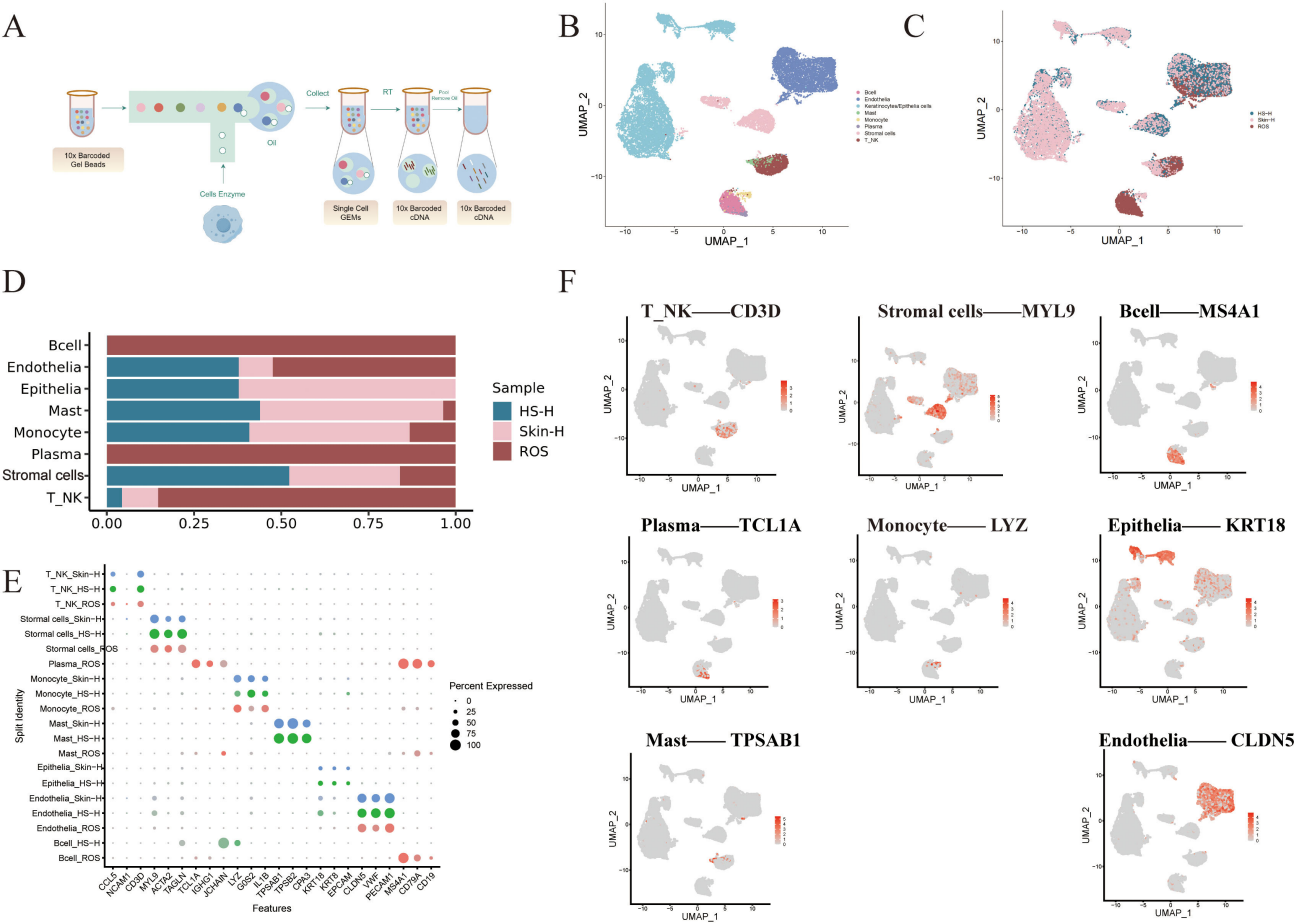
Single-cell analysis of skin conditions. **(A)** Flowchart of the study. **(B, C)** UMAP plot of different cell subtypes and distribution in Skin-H, HS-H and ROS. **(D)** The proportion of each cluster in Skin-H, HS-H and ROS. **(E)** Quantified the proportions and lineage-specific marker genes of eight lineages- B lymphocytes, endothelial, epithelial, mast, monocyte, plasma, stromal cells, and T cells—across Skin-H, HS-H and ROS. **(F)** Feature plots of expression distribution for selected cluster-specific genes. The scRNA-seq data visualized in this figure are derived from one individual sample per condition (healthy skin, hypertrophic scar, and rhinophyma).

Single-cell dissociation was performed on three tissue samples (one each from healthy skin, hypertrophic scar, and rhinophyma), followed by scRNA-seq (**Figure 1A**). This analysis therefore compares data from one individual per condition (N = 1). After quality control, we obtained transcriptomic information from 28,753 cells (healthy skin: 10,189; hypertrophic scar: 10,429; rhinophyma: 8,072) and constructed a gene expression matrix for each cell. The subjects were categorized into three distinct groups: the healthy skin group (Skin-H), the hypertrophic scar group (HS-H), and the rhinophyma type rosacea group (ROS). Following filtration, normalization, and dimensionality reduction, we conducted unsupervised deep embedding clustering and visualized these 28,753 cells using UMAP (**Figures 1B, C**) and categorized them into eight cellular lineages based on lineage-specific marker genes (**Figure 1D**), and quantified the proportion of these eight lineages across the three tissues, including B lymphocytes (B cells; labeled by MS4A1, CD79A, and CD19), endothelial cells (Endothelia; labeled by CLDN5, VWF, and PECAM1), epithelial cells (Epithelia; labeled by KRT18, KRT8, and EPCAM), mast cells (Mast; labeled by TPSAB1, TPSB2, and CPA3), monocyte cells (Monocyte; labeled by LYZ, G0S2, and IL1B), plasma cells (Plasma; labeled by TCL1A, IGHG1, and JCHAIN), smooth muscle cells (SMC; labeled by MYL9, ACTA2, and TAGLN), and T cells (T-NK; labeled by CD3D, CCL5, and NCAM1) (**Figure 1E**). These clusters exhibited distinct molecular characteristics (**Figure 1F**), reflecting the cellular diversity and heterogeneity of healthy skin, hypertrophic scar, and rhinophyma. The UMAP plot displayed the distribution of major marker genes of the eight cell types across the three tissues. B cells were found exclusively in rhinophyma; endothelial cells were present in all three tissues, with a notable abundance in hypertrophic scar compared to healthy skin; epithelial cells were observed only in healthy skin and were absent in rhinophyma; mast cells were more abundant in hypertrophic scar than in rhinophyma; monocyte cells were more prevalent in hypertrophic scar than in rhinophyma; plasma cells were found only in rhinophyma; smooth muscle cells were more abundant in healthy skin than in rhinophyma; and T-NK cells were more abundant in rhinophyma than in healthy skin (**Figures 1B–D**). We further analyzed these five cell types to explore the fibrotic microenvironment differences between hypertrophic scars and rhinophyma.

### 3.2 Endothelial cell subtypes analysis

**Figure 2:**
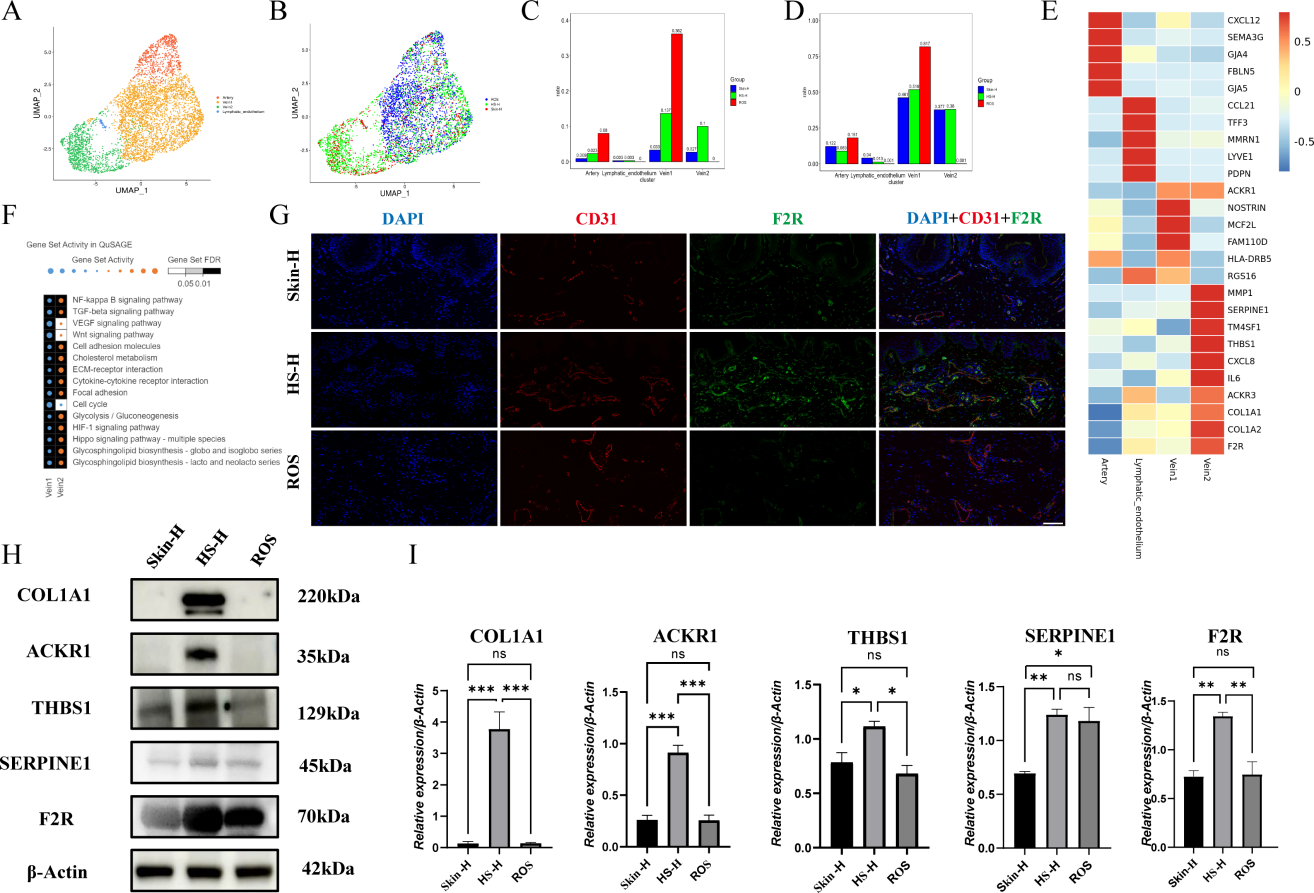
Endothelial cell subtypes analysis. **(A, B)** UMAP plot of different endothelial cell subtypes and distribution in Skin-H, HS-H and ROS. **(C, D)** The proportion of each endothelial subtype in Skin-H, HS-H and ROS. **(E)** Heat map of correlation between differentially expressed genes. **(F)** Gene set activity and differences in gene pathways in Vein1 and Vein2. The significance threshold was set to an adjusted P-value < 0.05. **(G)** Images from Western blot analysis displaying the protein expression levels of five genes linked to endothelial cell function. **(H)** CD31 and F2R immunofluorescence in Skin-H, HS-H and ROS tissues. All panels are at 20× magnification. **(I)** The western blot assay of COL1A1, ACKR1, THBS1, SERPIME1 and F2R in Skin-H, HS-S and ROS tissues. Data are mean ± SD of triplicate measurements normalized to β-Actin. Statistical significance was determined by Student’s t-test, *P < 0.05, **P < 0.01, ***P < 0.001 and ns means no statistical difference.

Endothelial cells were categorized into four distinct subtypes, specifically arterial, venous 1, venous 2, and lymphatic endothelial cells, as illustrated in clusters in **Figures 2A, B**. Compared to normal skin (Skin-H) and hypertrophic scar (HS-H) samples, Vein1 is significantly increased in Rhinophyma (ROS) samples (**Figures 2C, D**). Vein1 mainly highly expresses marker genes such as NOSTRIN, MCF2L, and FAM110D. Vein2 mainly highly expresses marker genes such as MMP1, SERPINE1, TM4SF1, THBS1, CXCL8, IL6, ACKR3, COL1A1, and COL1A2. Artery mainly highly expresses marker genes such as CXCL12, SEMA3G, GJA4, FBLN5, and GJA5. Lymphatic-endothelium mainly highly expresses genes such as CCL21, TFF3, MMRN1, LYVE1, and PDPN (**Figure 2E**). Vein1 can be defined as a normal type of endothelial cell, whereas Vein2 represents a distinct endothelial cell type that exhibits elevated collagen expression and participates in pro-inflammatory and pro-tumorigenic processes. Vein2 outperforms Vein1 in extracellular matrix, angiogenesis, inflammation, and glucose metabolism. Using the QuSAGE method, we analyzed gene set activity in Vein1 and Vein2 to uncover differences in gene pathways. In Vein2, pathways such as NF-κB, TGF-β, cell adhesion, cholesterol metabolism, ECM-receptor interaction, cytokine interaction, focal adhesion, glycolysis, HIF-1, Hippo signaling, and glycosphingolipid biosynthesis are activated. This suggests Vein2 has unique functions in cell signaling, interaction, metabolism, cytoskeletal reorganization, and survival (**Figure 2F**). Western blot analyses reveal that in HS-H, proteins associated with Vein2, including COL1A1, THBS1, ACKR1, Serpine1, and F2R, exhibit elevated expression levels. This suggests the presence of a vein subtype in HS-H that facilitates fibrosis, promotes angiogenesis, and contributes to the remodeling of the extracellular matrix (**Figures 2G, I**). In addition, immunofluorescence analysis revealed that the distribution of CD31 and F2R showed that the vascular density in ROS was higher compared to HS-H, characterized by marked vessel dilation and a pronounced expression of F2R protein surrounding the vessels in HS-H (**Figure 2H**). Endothelial cell abnormalities may play a critical role in the formation of HS-H. Conversely, the dilation of blood vessels and adequate oxygen levels in ROS could contribute to scar-free healing following scratch surgery in ROS.

### 3.3 Vein1-Vein2 transition mechanisms

**Figure 3:**
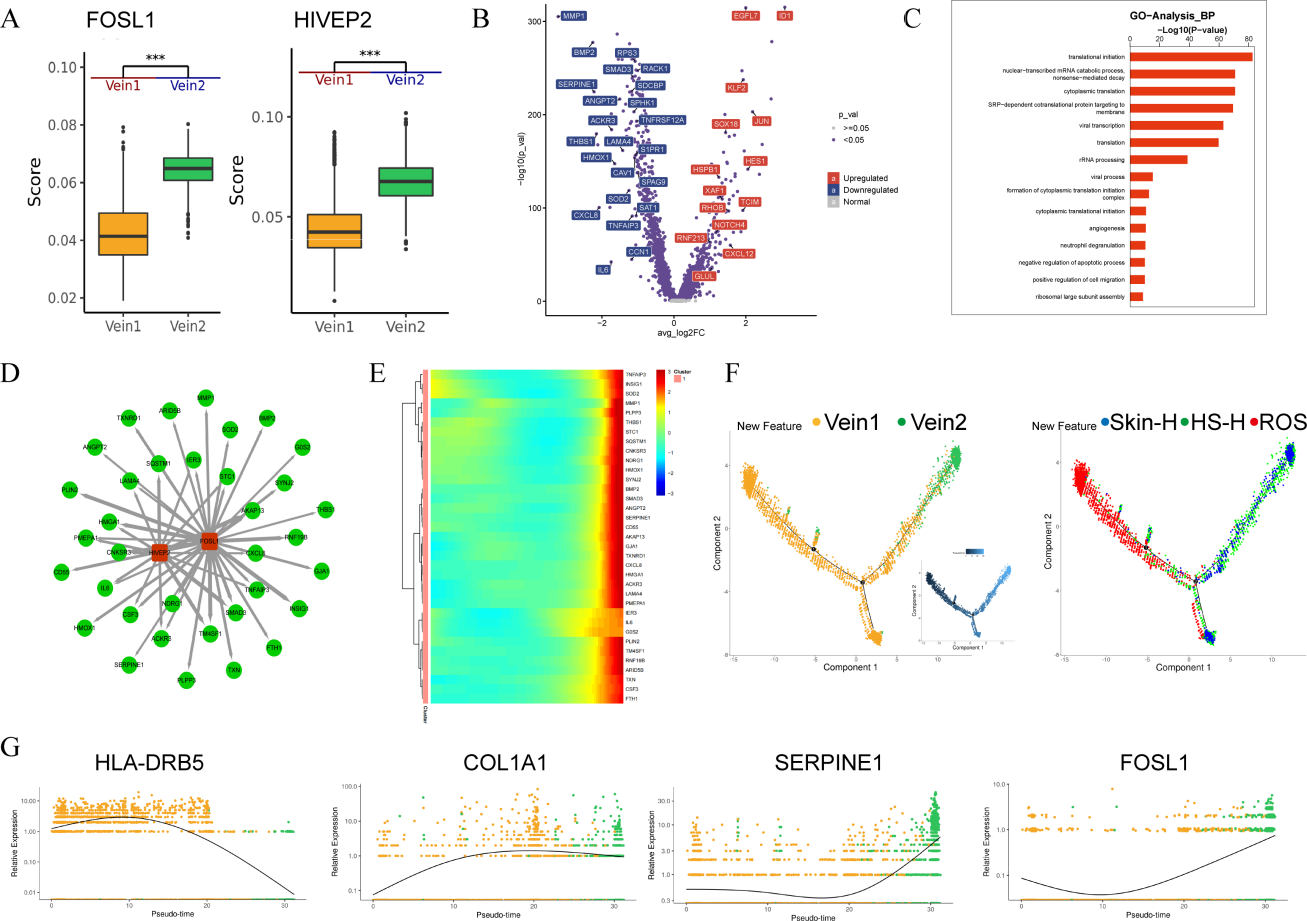
Vein1-Vein2 transition mechanisms. **(A)** The score of FOSL1 and HIVEP2 in Vein1 and Vein2 subtypes. **(B)** Differences in gene expression between Vein-1 and Vein-2 populations are depicted on a volcano map, red indicates up-regulated gene, which is highly expressed in Vein1, blue indicates down-regulated gene, which is highly expressed in Vein2. **(C)** GO analysis for Vein2's biological processes. **(D)** The target genes of FOSL1 and HIVEP2 which linked to Vein2 markers. **(E)** The heatmap of genes highly expressed in Vein2. **(F)** Trajectory plot showing the pseudo-time trend. As the color became lighter, pseudo-time gradually marched. The left trajectory plot shows location and distribution of different cell types. The right trajectory plot shows different states in different tissue types. **(G)** Scatterplot showing the pseudo-time differences in gene expression of HLA-DRB5, COL1A1, SERPINE1 and FOSL1, with solid lines representing from Vein1 to Vein2 branching direction. ***p < 0.001.

To explore the reasons for the presence of two distinct subtypes, Vein1 and Vein2, in HS-H and ROS, we conducted an analysis of the transcription factors in Vein1 and Vein2. The results showed that FOSL1 and HIVEP2 are highly expressed in Vein2 (**Figure 3A**). To further characterize the functional differences between Vein1 and Vein2, we visualized the differential genes of Vein1 and Vein2 using a volcano plot (**Figure 3B**). The results indicated that genes upregulated in Vein1 include EGFL7 and ID1. Genes upregulated in Vein2 include SERPINE1 and TM4SF1. We performed Gene Ontology (GO) analysis for Vein2’s biological processes (BP) (**Figure 3C**). Notably, we found that translational initiation, nuclear transcribed mRNA catabolic process, nonsense-mediated decay, cytoplasmic translation, and SRP-dependent cotranslational protein targeting to membrane were activated in Vein2. Our Sceinc analysis revealed that the target genes of FOSL1 and HIVEP2 are linked to Vein2 markers, suggesting that these genes might affect the ultimate development of Vein2 (**Figure 3D**). The heatmap of genes highly expressed in Vein2 during this process (**Figure 3E**). In summary, FOSL1 and HIVEP2 not only facilitate the creation of abnormal endothelium but also significantly increase the fibrotic potential in HS-H tissue compared to ROS, potentially explaining the differing fibrosis mechanisms in HS-H and ROS.

To investigate the relationship and regulatory dynamics between Vein1 and Vein2 cell phenotypes, we used Monocle2 to identify a branching trajectory with two main branches. Branch 1 is primarily composed of endothelial cells from ROS, representing the Vein1 phenotype, while branch 2 mainly consists of Skin-H and HS-H cells, ending with the Vein2 phenotype. This suggests distinct endothelial cell states in Skin-H, HS-H, and ROS. Vein1 is predominant at the start of the trajectory, indicating the initial endothelial state, while Vein2 is mainly at the end, indicating the final state, with Skin-H cells positioned in between (**Figure 3F**).

During the transition from Vein1 to Vein2, the expression of HLA-DFB5 decreases, while the expression of COL1A1, SERPINE1, and FOSL1 increases (**Figure 3G**). HLA-DRB5 is a gene in the human leukocyte antigen system, primarily involved in antigen presentation processes within the immune system. The expression of COL1A1, SERPINE1, and FOSL1 represents the activation of matrix deposition and fibrotic processes. Overall, compared to Vein1, Vein2 exhibits increased immune suppression and collagen secretion, as well as the activation of epithelial-mesenchymal transition (EMT).

### 3.4 Stromal cell subtypes analysis

**Figure 4:**
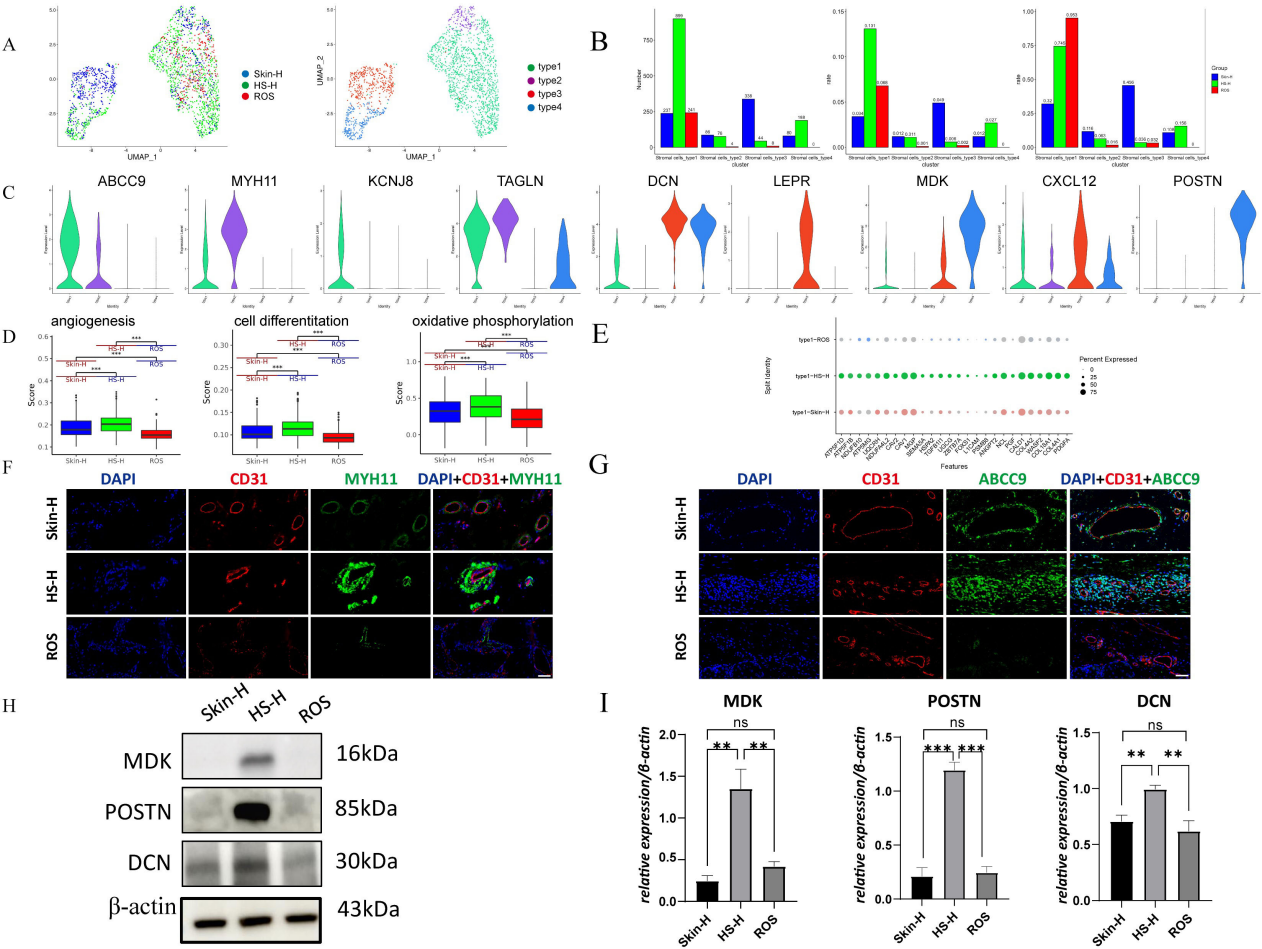
Stromal cell subtypes analysis. **(A)** UMAP plot of different stromal cell subtypes (Stromal cells_type1, Stromal cells_type2, Stromal cells_type3, and Stromal cells_type4) and distribution in Skin-H, HS-H and ROS. **(B)** The proportion of each stromal subtype in Skin-H, HS-H and ROS. **(C)** The expression level of marker genes for four stromal cell types. **(D)** The angiogenesis, cell differentiation and oxidative phosphorylation functions of stromal cells in Skin-H, HS-H, and ROS. **(E)** The relative expression percent of Stromal cells_type1 related genes in Skin-H, HS-H and ROS. **(F)** CD31 and MYH11 immunofluorescence in Skin-H, HS-H and ROS tissues. **(G)** CD31 and ABCC9 immunofluorescence in Skin-H, HS-H and ROS tissues. All panels are at 40× magnification. **(H, I)** The expression of MDK, POSTN, DCN in Skin-H, HS-S and ROS tissues. Data are mean ± SD of triplicate measurements normalized to β-Actin. Statistical significance was determined by Student’s t-test, **P < 0.01, ***P < 0.001 and ns means no statistical difference.

scRNA-seq was utilized to dissect the cellular composition of stromal cells, revealing distinct subtypes within the HS-H, ROS, and Skin-H groups (cells from clusters SMC_type1, SMC_type2, SMC_type3, and SMC_type4 are shown in **Figure 4A**, further identifying four unique subtypes (**Figures 4A, B**). **Figure 4B** presents the following information from left to right: the number of cells measured for each type, the proportion of different cell types among all stromal cells, and the proportion of various cell types within the stromal cells of each tissue. SMC_type1 expresses high levels of ABCC9 and KCNJ8 and is defined as angiogenesis-promoting pericytes. SMC_type2, absent in the ROS group, expresses MYH11 and TAGLN, characterizing a specific smooth muscle cell. SMC_type3, abundant in the Skin group, expresses DCN, LEPR, and CXCL12, identifying it as a mesenchymal-like fibroblast with stem cell traits. SMC_type4, prevalent in the HS-H group, expresses MDK and POSTN and is recognized as a mature fibroblast that secretes large amounts of collagen (**Figure 4C**).

By analyzing the functions of stromal cells in Skin-H, HS-H, and ROS, we found that angiogenesis, cell differentiation, and oxidative phosphorylation are most robust in HS-H, followed by Skin-H, and weakest in ROS (**Figure 4D**). The relative expression percent of SMC_type1 related genes in the three groups that the expression levels of NDUFB10, ATP5MG, and HSD17B2 were higher in the HS-H group compared to the Skin-H group, indicating that SMC_type1 cells in HS-H tissues exhibit enhanced energy metabolism (**Figure 4E**). Consequently, we performed immunofluorescence staining for SMC_type1 and SMC_type2 (**Figures 4F, G**), using ABCC9 to mark pericytes, MYH11 to mark smooth muscle cells, and CD31 to mark blood vessels. The results indicate that in HS-H, pericytes and smooth muscle cells are abundant and extensively envelop blood vessels, potentially causing tissue hypoxia (15, 16), exacerbating the hypoxic environment in HS-H. However, in ROS, blood vessels are loosely structured and lack the enclosure of pericytes and smooth muscle. Western blot results indicate that SMC_type3 cells (DCN-marked) are most prevalent in HS-H, with similar levels in ROS and Skin-H, while SMC_type4 cells (marked by MDK and POSTN) are mainly found in HS-H and less so in ROS and Skin-H (**Figures 4H, I**). The variance between mesenchymal-like and mature fibroblasts could significantly influence fibrosis levels in HS-H and ROS.

### 3.5 Fibroblast subtype differences

**Figure 5:**
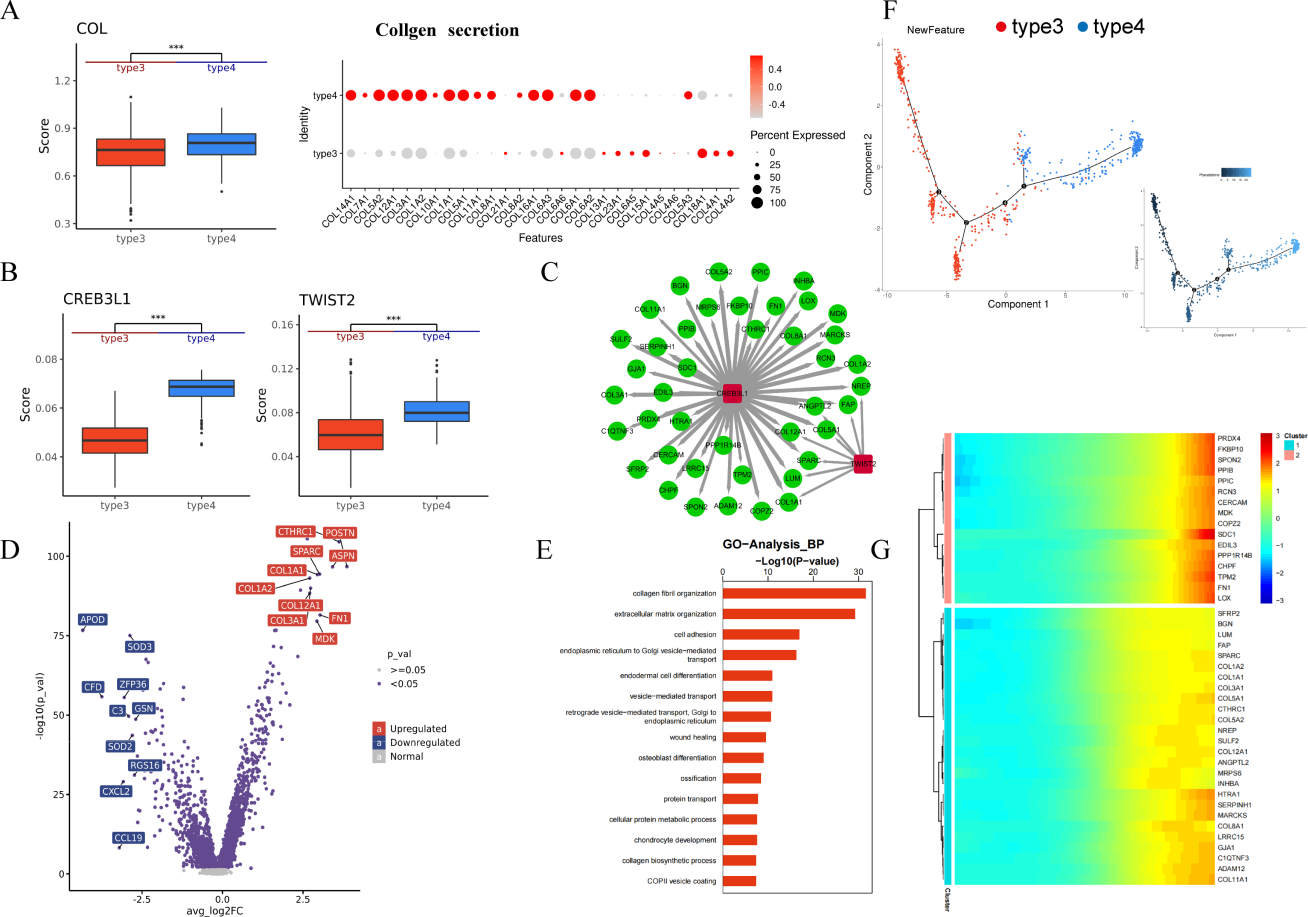
Gene expression and diffusion-pseudotime analysis of Stromal cells_type3 and Stromal cells_type4. **(A)** In scores of collgen secretion function and the specific secretion levels of each type of collagen of Stromal cells_type3 and Stromal cells_type4. **(B)** Estimated CREB3L1 and TWIST2 scores of Stromal cells_type3 and Stromal cells_type4. **(C)** The weighted gene co-expression network map showed the genes that were correlated with CREB3L1 and TWIST2, and the strength of the relationship was indicated by the thickness of the connection. **(D)** Differences in gene expression between Stromal cells_type3 and Stromal cells_type4 populations are depicted on a volcano map, red indicates up-regulated gene, which is highly expressed in Stromal cells_type4, blue indicates down-regulated gene, which is highly expressed in Stromal cells_type3. **(E)** GO analysis of Stromal cells_type3 and Stromal cells_type4's biological functions. **(F)** The diffusion-pseudotime analysis of type3 and type4, colored by subcluster. **(G)** The cluster analysis of two pseudotime cell populations, during the differentiation of type3 into type4, genes related to fibrosis promotion, the change in collagen production, extracellular matrix deposition, cell proliferation, genes associated with immune suppression, cell proliferation, and migration expressed (***P < 0.001).

To explore the differences between SMC_type3 and SMC_type4 in HS-H, we first analyzed their collagen secretion profiles. SMC_type4 exhibits a greater capacity for collagen secretion compared to SMC_type3, with both the types and expression levels of collagen secreted by SMC_type4 being higher than those of SMC_type3 (**Figure 5A**). We examined the transcriptional regulatory networks in HS-H formation, finding that CREB3L1 and TWIST2 are crucial transcription factors in HS-H myofibroblasts (**Figure 5B**). Notably, scRNA-seq studies have consistently identified CREB3L1 as a key factor in fibrotic skin diseases (17). Moreover, TWIST1, a key paralog of TWIST2, plays a role in hypertrophic scar fibrosis and could be a therapeutic target, indicating TWIST2 might also be a viable target for HS-H treatment (18, 19). Through SCENIC analysis, we uncovered the significant roles of transcription factors CREB3L1 and TWIST2 in cellular regulatory networks (**Figure 5C**). CREB3L1 target genes are linked to ECM formation, cell growth, signal transduction, and stress responses, especially in scar and tissue remodeling. TWIST2 target genes are vital for tissue structure maintenance, cell proliferation and migration, and tissue repair and remodeling. We presented the differential genes between SMC_type3 and SMC_type4 in a volcano plot format (**Figure 5D**), showing that genes promoting fibrosis such as CTHRC1, POSTN, SPARC, ASPN, FN1, MDK and related to collagen secretion in COLs are highly expressed in SMC_type4; whereas in SMC_type3, antioxidant genes such as APOD, SOD2, and SOD3, inflammation regulation and immune genes: CFD, CXCL2, CCL19, RGS16, and cell protection and regulatory genes: ZFP36, GSN are highly expressed. We examined the biological functions of SMC_type4 and SMC_type3 (**Figure 5E**). SMC_type4 shows enhanced activity in collagen fibril and ECM organization compared to SMC_type3, suggesting it is more effective in promoting fibrosis and extracellular matrix formation. To further explore the relationships between fibroblast subtypes and investigate the regulatory dynamics during the phenotypic transition from SMC_type3 to SMC_type4, we used Monocle2 to reveal a branching trajectory with two main branches, where SMC_type3, over time, partially transforms into SMC_type4, while some retain their original SMC_type3 phenotype (**Figure 5F**).

We performed cluster analysis on the two pseudotime cell populations (**Figure 5G**), and the results showed that during the differentiation of type3 into type4, genes related to fibrosis promotion, collagen production, extracellular matrix deposition, and cell proliferation, as well as genes associated with immune suppression, cell proliferation, and migration, are highly expressed. Overall, type3 is a type of fibroblast with anti-fibrotic capabilities; however, during the differentiation process towards type4, the anti-fibrotic ability gradually diminishes, and instead, these cells become more prone to secreting collagen, promoting extracellular matrix deposition, and accelerating the fibrosis process.

### 3.6 Epithelial cell abnormalities in HS-H

**Figure 6:**
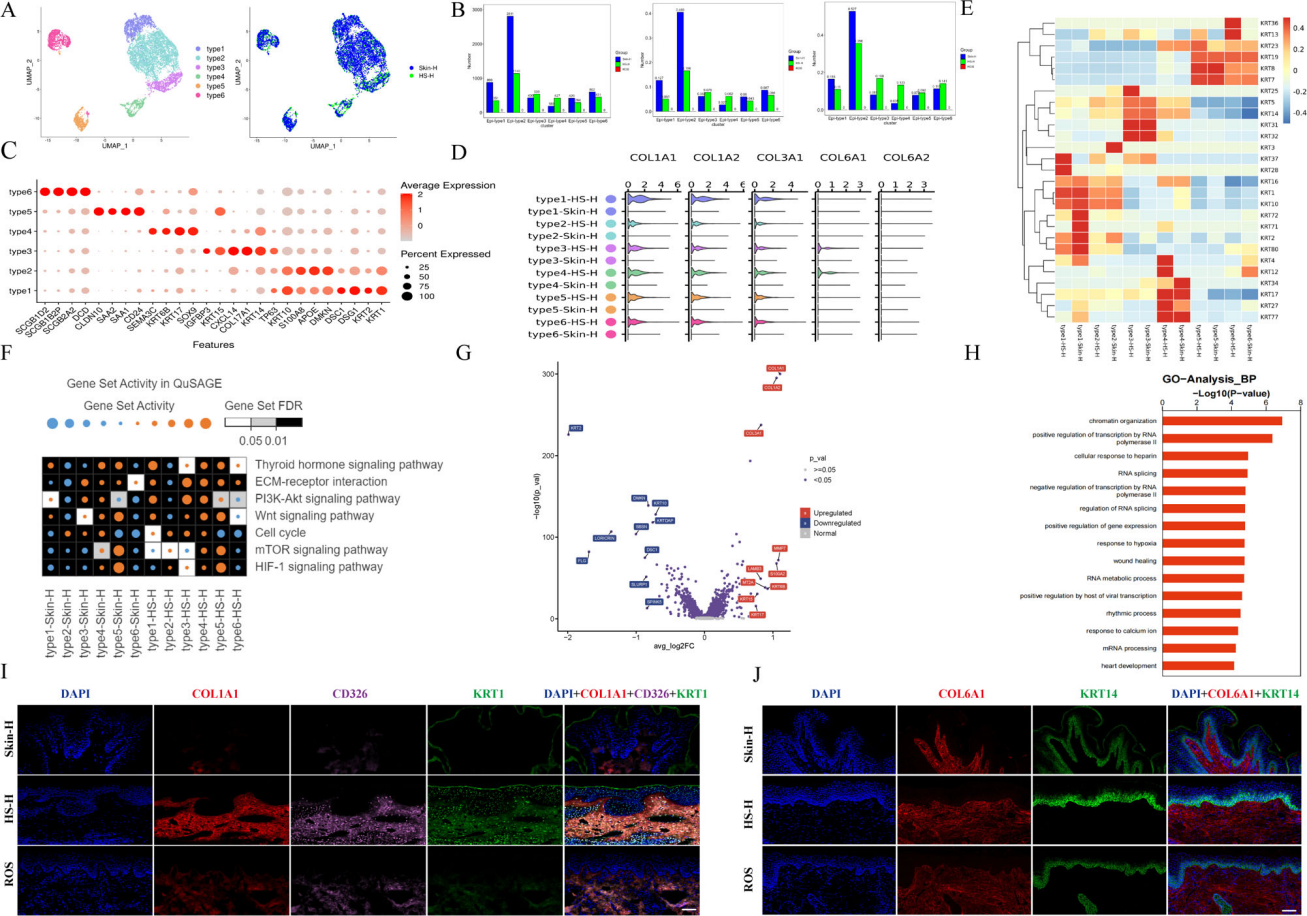
The differences of epithelial cells in HS-H and Skin-H. **(A)** UMAP plot of different epithelial cell subtypes and distribution in Skin-H and HS-H. **(B)** The proportion of each epithelial subtype in Skin-H and HS-H. **(C)** The relative expression gene percent of these subgroups. **(D)** The expression of collagen genes in these six types of epithelial cells. **(E)** Heat map of correlation between differentially expressed genes. **(F)** Gene set activity and differences in gene pathways in Skin-H and HS-H. The significance threshold was set to an adjusted P-value < 0.05. **(G)** Differences gene expression of keratinocytes between Skin-H and HS-H populations are depicted on a volcano map, blue color display up-regulated genes in Skin-H, red color display up-regulated genes in HS-H. **(H)** The GO analysis of upregulated biological processes in HS-H compared to Skin-H. **(I)** COL1A1 and CD326 immunofluorescence in Skin-H, HS-H and ROS tissues. **(J)** COL6A1 and KRT14 immunofluorescence in Skin-H, HS-H and ROS tissues. All panels are at 20× magnification.

To elucidate the significant differences in epithelia cells between HS-H and ROS, and to understand the role of epithelia cells in the pathogenesis of fibrosis, we conducted an unsupervised clustering analysis of keratinocytes from HS-H and Skin-H. Hierarchical clustering analysis revealed that epithelial cells derived from HS-H and Skin-H were divided into six subgroups, with an increase in type3 and type4 in HS-H being the focus of this study (**Figures 6A, B**). We further explored the different expressed genes among these subgroups, which included: Type1: KRT1, KRT2, DSG1, DSC1; Type2: DMKN, APOE, S100A8, KRT10; Type3: TP63, KRT14, CXCL14, KRT15; Type4: SOX9, KRT17, KRT6B, SEMA3C; Type5: CD24, SAA1, SAA2, CLDN10; Type6: DCD, SCGB2A2, SCGB1B2P, SCGB1D2 (**Figure 6C**). Type3 epithelial cells mainly express basal cell markers TP63, KRT14, KRT15 (20). The epithelial-derived CXCL14 primarily inhibits tumor progression (21), indicating that Type3 is a category of epithelial cells with stem or progenitor cell characteristics, possessing the potential for multidirectional differentiation. We also analyzed the expression of collagen genes in these six types of epithelial cells (**Figure 6D**). Epithelial cells from Skin-H do not express collagen genes, while Type1–6 from HS-H express COL1A1, COL1A2, COL3A1; Type3 and Type4 only express COL6A1 and COL6A2. Compared to Skin-H, Type3 in HS-H highly expresses KRT14 and KRT15, and Type4 highly expresses KRT4 and KRT12 (**Figures 6C, E**). KRT15 is a marker of epidermal stem cells (22). The high expression of KRT15 may also represent an imbalance in the basal-like cell to epidermal differentiation program, potentially indicating a state of imbalanced differentiation of stem-like keratinocytes (23). In type3-HS-H, ECM-receptor interaction and PI3K-Akt signaling pathways are more activated than in type3-Skin-H. In type4-HS-H, ECM-receptor interaction, PI3K-Akt, and Wnt signaling pathways show increased activation compared to type4-Skin-H. (**Figure 6F**). We analyzed the differential genes of keratinocytes in Skin-H and HS-H, with the results represented in a volcano plot (**Figure 6G**), indicating that keratinocytes in Skin-H mainly express genes such as LORICRIN, FLG, SLURP1, KRT2, KRT10, which are related to skin barrier function, cell differentiation, immune regulation, and cellular stress response, representing a group of normal keratinocytes. Keratinocytes in HS-H mainly express genes such as COL1A2, MMP7, LAMB3, MT2A, KRT15, COL3A1, S100A2, whose expression is associated with collagen deposition, inflammatory response, tissue repair, and apoptosis. The upregulated GO analysis in the group of hypertrophic scar compared to the group of normal skin (**Figure 6H**) shows that biological processes such as chromatin organization and positive regulation of transcription by RNA polymerase II are elevated in the scar group, which may represent active biological processes in gene expression regulation and chromatin structure. Immunofluorescence staining revealed that COL1A1, CD326, and KRT1 are highly expressed in HS-H compared to ROS and Skin-H. CD326 is linked to HS-H recurrence, while KRT1, typically found in the epidermis, is abnormally distributed in both the epidermis and dermis of HS-H, indicating keratinocyte proliferation. KRT14, a skin stem cell marker, is present in the stratum corneum of all three tissues, with a thicker layer and inward-growing keratinocytes in HS-H. COL6A1 is expressed in all tissues (**Figures 6I, J**). These findings highlight keratinocyte abnormalities as crucial to HS-H pathogenesis.

### 3.7 Immune cell dynamics

**Figure 7:**
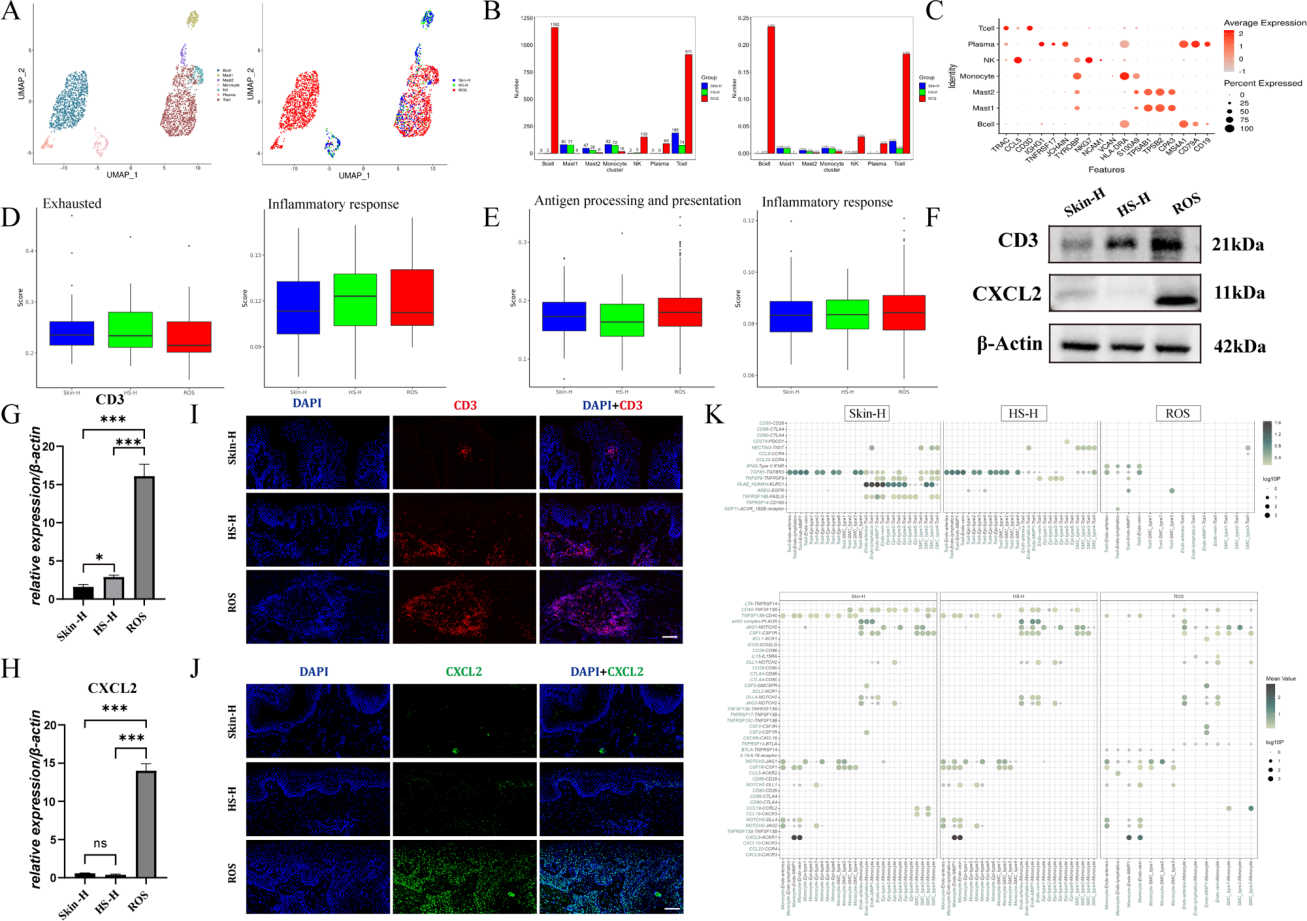
Immune cell dynamics. **(A)** UMAP plot of different immune cell subtypes (B cells, Mast1, Mast2, Monocytes, NK cells, Plasma cells, and T cells) and distribution in Skin-H, HS-H and ROS. **(B)** The proportion of each immune cell subtype in Skin-H, HS-H and ROS. **(C)** The expression level of marker genes for seven immune cell subtypes. **(D)** The inflammatory response scores associated with exhaustion functions scores in Skin-H, HS-H, and ROS. **(E)** The assessment of monocyte functions revealed that capabilities in antigen processing with presentation and in mediating inflammatory responses scores in Skin-H, HS-H, and ROS. **(F–H)** The expression of CD3, CXCL2 in Skin-H, HS-S and ROS tissues. Data are mean ± SD of triplicate measurements normalized to β-Actin. Statistical significance was determined by Student’s t-test, ***P < 0.001 and ns means no statistical difference. **(I, J)** CD3 and CXCL2 immunofluorescence in Skin-H, HS-H and ROS tissues. All panels are at 20× magnification. **(K)** Cellphone DB analysis results to demonstrate receptor-ligand interactions between different cell types in Skin-H, HS-H, and ROS.

We classified immune cells into seven types: B lymphocytes (B cells), Mast1, Mast2, Monocytes, NK cells, Plasma cells, and T cells; their distribution across three tissues is displayed through UMAP plots (**Figure 7A**). In ROS, the presence of B cells, NK cells, Plasma cells, and T cells is higher than in HS-H and Skin-H (**Figure 7B**). B cells: labeled by MS4A1 and CD79A, Mast1 (Mast; labeled by TPSAB1, TPSB2, and CPA3), Mast2 (Mast; labeled by S100A9, TPSAB1, TPSB2, and CPA3), Monocyte cells (Monocyte; labeled by TYROBP, HLA-DRA, and S100A9), Natural Killer cells (NK; labeled by CCL5 and NKG7), Plasma cells (Plasma; labeled by MS4A1, CD79A and CD19), T cells (T-NK; labeled by CD3D and TRAC) (**Figure 7C**). We conducted an evaluation of T cell functions and observed that T cells in the HS-H group demonstrated a more pronounced state of exhaustion relative to the other two groups. This was accompanied by an elevated tissue inflammatory response associated with exhaustion, as illustrated in **Figure 7D**. Furthermore, our assessment of monocyte functions revealed that monocytes in the ROS group displayed enhanced capabilities in antigen processing and presentation, as well as in mediating inflammatory responses, when compared to the other two groups (**Figure 7E**). However, it is important to note that the differences in cell function scores among the three groups did not reach statistical significance.

Immunofluorescence and western blot results show (**Figures 7F–J**): the high expression of CXCL2 and CD3 in ROS leads to the recruitment of immune cells, which may be one of the reasons why rhinophyma does not progress to hypertrophic scars after surgery. T cell infiltration in ROS is significantly higher than in HS-H and Skin-H, which may be related to the leaky vascular structure in ROS.

We used Cellphone DB analysis results to demonstrate receptor-ligand interactions between different cell types (**Figure 7K**). In Skin-H, TGF-β1 produced by T cells can bind to TGF-βR3 expressed by Endo-arteries, Endo-lymphatics, Endo-MMP1, Endo-vein, Epi-type2, Epi-type3, Epi-type5, Epi-type6, SMC-type3, and SMC-type4, HLAE-HUMAN produced by Endo-arteries, Endo-lymphatics, Endo-MMP1, Endo-vein, Epi-type1, Epi-type2, Epi-type3, Epi-type4, SMC-type2, and SMC-type3 can bind to KLRC1 expressed by T cells (The left column in **Figure 7K**). In HS-H, the communication strength of TGF-β1 produced by T cells with TGF-βR3 of other cells is lower than in Skin-H but higher than in ROS, the communication strength of HLAE-HUMAN produced by other cells with KLRC1 of T cells is lower than in Skin-H but higher than in ROS (The middle and right columns in **Figure 7K**). This indicates that NKG2A, encoded by the KLRC1 gene, is a primary NK inhibitory receptor, and high expression and activation of NKG2A in T cells represent a weaker immune function in HS-H compared to ROS (24, 25).

## 4 Discussion

Previous single-cell sequencing studies often compared normal skin with hypertrophic scar (HS) to identify potential therapeutic targets for HS. By including ROS tissue as a control, we aim to explore the mechanisms of scar and rhinophyma formation from a new perspective, in hopes of finding new targets for scar therapy and understanding the principles behind the normal healing post-rhinophyma surgery.

One of the most intriguing discoveries is that the endothelial cell landscape, we identified an abnormal endothelial cell subtype, Vein2, in HS-H tissue, which highly expresses COL1A1, MMP1, ACKR1, THBS1, SERPINE1, and F2R. Additionally, the transcription factors FOSL1 and HIVEP2 are highly expressed in Vein2. Studies have shown that FOSL1 protein is overexpressed and more stable in hypertrophic disorders and fibromatoses, and the FOS gene family is closely related to the occurrence of mesenchymal tumors (26). HIVEP2 has a close relationship with TGF-β, and the activation of the TGF-β signaling pathway can promote the expression of HIVEP2. In the absence of HIVEP2, TGF-β signaling is terminated, and the presence of HIVEP2 can protect phosphorylated Smad2 and Smad3 from dephosphorylation by nuclear phosphatases. This leads to the sustained activity of phosphorylated Smad proteins in signal transduction (27). Overall, FOSL1 and HIVEP2 not only promote the generation of abnormal endothelium but also enhance the fibrotic capacity in HS-H tissue far above that in ROS, which may be the mechanism by which fibrosis occurs differently in HS-H and ROS. In Vein1, EGFL7 and ID1 are elevated; in Vein2, SERPINE1 and TM4SF1 are elevated. EGFL7 can control vascular development and integrity and can also reduce the expression of COL1A1 (28, 29). ID1 can inhibit TGF-β-induced collagen expression (30). SERPINE1 can lead to thrombosis by inhibiting plasminogen activation and fibrin degradation; the expression of the SERPINE1 gene is highly induced by TGF-β, and Smad proteins are also involved in TGF-β-induced SERPINE1 transcription (31). TM4SF1 can promote the expression of VEGF-A and E-cadherin and can promote EMT and angiogenesis (32). This suggests that the vascular structure in ROS is more intact and has a certain inhibitory effect on collagen expression; the vessels in HS-H are more prone to thrombosis and have a strong pro-fibrotic effect. Considering the critical role of blood vessels in wound healing, an unanticipated discovery was the pronounced tendency of vessels in HS-H to form thrombi and facilitate fibrosis, which contributes to postoperative recurrence. In contrast, ROS demonstrates normal skin healing following rhinophyma surgery.

In the matrix cell landscape, we found that pericytes (SMC_type1) and smooth muscle cells (SMC_type2) are abundant in HS-H and wrap around blood vessels; in ROS, pericytes and smooth muscle cells are absent outside the vessels. These results further support the cause of hypoxia in HS-H tissue, and a hypoxic environment will enhance the angiogenesis function of HS-H (15); previous studies have demonstrated that, the enhancement of oxidative phosphorylation will also increase oxygen consumption (16), leading to worsening hypoxia in HS-H. However, this result has not previously been described that in ROS, the blood vessels are loose and lack the wrapping of pericytes and smooth muscle cells, and such leaky blood vessels may lead to the leakage of immune cells, resulting in a large infiltration of immune cells in ROS. This intriguing finding may be attributed to the absence of mature fibroblasts (SMC_type1) in ROS tissue, suggesting that the development of mature fibroblasts might be inhibited during ROS formation. This inhibition could potentially explain the normal healing observed following rhinophyma surgery. Conversely, the proportion of such cells in normal skin is relatively high, indicating that SMC_type4 cells are also present in normal skin. Typically, injuries to the dermis of normal skin result in scarring; however, when rhinophyma is completely excised, scarring does not occur. This phenomenon may be associated with the presence of this particular cell type. By analyzing the functions of matrix cells, we found that angiogenesis, cell differentiation, and oxidative phosphorylation are strongest in HS-H, followed by Skin-H, and weakest in ROS. This is consistent with previous studies, which found that compared to normal skin, hypertrophic scar has an increased number of vessels with more dilated phenotypes (33). Oxidative phosphorylation is an efficient way to generate the cellular energy currency ATP in a cascade of redox reactions. When oxidative phosphorylation function is enhanced, cells consume more oxygen to maintain efficient ATP production, which usually occurs in cells in a high metabolic state, such as muscles in intense exercise and rapidly dividing cells (34). This may indicate that the matrix cells in HS-H are in a high-energy metabolic state. Fibroblasts differentiate into myofibroblasts and express α-SMA, which makes the wound contract and is an important part of hypertrophic scar formation (35).

In the context of the epithelial cell landscape, our findings indicate an increase in type 3 and type 4 epithelial cells in HS-H. Type 3 epithelial cells predominantly express basal cell markers such as TP63, KRT14, and KRT15 (20), as well as CXCL14, suggesting that type 3 cells possess characteristics akin to those of keratinocyte stem or progenitor cells, with the capacity for multidirectional differentiation. In states of tissue damage or disease, these stem keratinocytes are capable of differentiating into various epithelial cell types, thereby facilitating the restoration of tissue function. Notably, in idiopathic pulmonary fibrosis (IPF), there is a marked abundance of basal cells (36). Additionally, type 3 cells express CXCL14, which has been shown to inhibit tumor progression (21), potentially elucidating the distinction between scars and tumors. Type 4 cells primarily express basal cell markers such as SOX9 and KRT17. SOX9 is critical for extracellular matrix (ECM) deposition across diverse tissues and organs (37). Recent research has demonstrated an upregulation of SOX9 transcription levels in the hearts of mice, which coincides with an increase in COL1A1 expression (38). Furthermore, studies indicate that the downregulation of SOX9 results in a reduction of cellular stemness (39). Recent single-cell RNA sequencing (scRNA-seq) analyses of IPF tissue have identified a distinct population of abnormal basal-like KRT17 cells characterized by dedifferentiation and profibrotic properties (40). SEMA3C has been shown to facilitate wound healing (41). This suggests that type 4 keratinocytes possess stem cell-like properties and contribute to both fibrosis and wound healing. This phenomenon may underlie the recurrence of keloid scar tissue following surgical intervention. Through immunofluorescence staining, we observed that keratinocytes expressing KRT1 exhibit disordered arrangements in hypertrophic scars, characterized by a scattered distribution throughout the tissue. In contrast, keratinocytes expressing KRT14 tend to proliferate inwardly. We hypothesize that the disorganization of keratinocytes in scars may result from aberrant epithelialization during the healing process. In the case of rhinophyma nodules, where no wound formation occurs, there is no epithelialization process, and consequently, no abnormal proliferation of keratinocytes is observed. Postoperative healing following rhinophyma surgery proceeds with normal epithelialization, possibly due to the absence of activated keratinocyte proliferation. In the context of the immune cell landscape, our findings indicate that the immune function in HS-H is comparatively weaker than in ROS. Notably, T cell infiltration is significantly greater in ROS than in both HS-H and Skin-H, potentially due to the leaky vascular structure characteristic of ROS. Furthermore, the pro-inflammatory cytokine CXCL2 is markedly overexpressed in rhinophyma, facilitating the recruitment of immune cells. These observations align with existing research, suggesting that the innate immune system in rhinophyma patients is perpetually activated and inflamed due to various triggering factors (42). Additionally, the polarization of M2 macrophages in HS-H contributes to immune suppression, thereby promoting the progression of HS-H formation (43).

Our research is subject to several limitations. Firstly, the study’s sample size was relatively small, and all samples were sourced from a single clinical center. This limitation affects the generalizability and representativeness of our findings. Secondly, while our preliminary analyses utilized bioinformatics approaches, such as cell temporal sequencing and communication analysis, to suggest potential mechanisms of cellular evolution and interaction, these findings necessitate further validation through animal experiments to establish causal relationships. For example, although we identified a potential role for FOSL1/HIVEP2 in the transformation of endothelial cell subtypes, this function has not been definitively characterized through gene intervention experiments. Consequently, our current conclusions should be regarded as preliminary. Future research should involve larger-scale, multi-center sample collections, coupled with both *in vitro* and *in vivo* functional experiments, to more robustly elucidate the underlying mechanisms and facilitate clinical translation.

## 5 Conclusion

In conclusion, our findings indicate that endothelial cells play a critical role in the progression of fibrosis associated with hypertrophic scar tissue. Additionally, the dysregulated proliferation of keratinocytes within this tissue may significantly contribute to the advancement of hypertrophic scar fibrosis. In the case of rhinophyma, the encasement of matrix cells around blood vessels not only influences the condition of these vessels but also facilitates the infiltration of immune cells due to the absence of pericyte and smooth muscle cell coverage. This immune cell infiltration may exert a cytotoxic effect on the abnormally proliferating keratinocytes, potentially serving as one of the mechanisms underlying normal healing following rhinophyma surgery. Postoperatively, targeting endothelial cells with anti-thrombotic treatments and keratinocytes with anti-proliferative therapies in hypertrophic scars may represent promising therapeutic strategies for preventing postoperative complications. Our findings reveal fundamental differences in the pre-operative state of these tissues, which we propose create a cellular context that is either permissive or resistant to fibrotic progression. The logical link lies in the well-established concept that the initial tissue milieu at the time of injury critically influences the healing outcome. However, it is important to note that our study is limited by a small sample size, necessitating further cases, samples, and replication studies to validate our conclusions.

## Data Availability

The original contributions presented in the study are included in the article/**Supplementary Material**. Further inquiries can be directed to the corresponding authors.
