# Supplementary material for: Distinct diversity of skin cell populations of rhinophyma and hypertrophic scar illustrated by scRNA-seq
Source: Front Immunol. 2026 Mar 12;17:1703469. doi: 10.3389/fimmu.2026.1703469 (PMC13017343; doi:10.3389/fimmu.2026.1703469)
Supplement: Supplementary file 1 [file Table1.docx]

**Supplementary Table 1. Patient and sample characteristics**

| **Sample ID** | **Age（years）** | **Sex** | **Race** | **Ethnicity** | **Anatomical**  **Site of Biopsy** | **Disease Status** | **scRNA-seq** | **Western**  **Blot** | **Immunofluorescence** |
| --- | --- | --- | --- | --- | --- | --- | --- | --- | --- |
| S1 | 30 | Man | Asian | Han Chinese | Prepuce | Healthy skin | Yes | Yes | Yes |
| S2 | 25 | Man | Asian | Han Chinese | Prepuce | Healthy skin | No | Yes | Yes |
| S3 | 41 | Man | Asian | Han Chinese | Prepuce | Healthy skin | No | Yes | Yes |
| S4 | 32 | Man | Asian | Han Chinese | Prepuce | Healthy skin | No | Yes | Yes |
| S5 | 34 | Man | Asian | Han Chinese | Prepuce | Healthy skin | No | Yes | Yes |
| S6 | 22 | Man | Asian | Han Chinese | Chest | Hypertrophic scar | Yes | Yes | Yes |
| S7 | 37 | Man | Asian | Han Chinese | Chest | Hypertrophic scar | No | Yes | Yes |
| S8 | 41 | Man | Asian | Han Chinese | Chest | Hypertrophic scar | No | Yes | Yes |
| S9 | 25 | Man | Asian | Han Chinese | Chest | Hypertrophic scar | No | Yes | Yes |
| S10 | 27 | Man | Asian | Han Chinese | Chest | Hypertrophic scar | No | Yes | Yes |
| S11 | 47 | Man | Asian | Han Chinese | Nasal skin | Rhinophyma | Yes | Yes | Yes |
| S12 | 56 | Man | Asian | Han Chinese | Nasal skin | Rhinophyma | No | Yes | Yes |
| S13 | 51 | Man | Asian | Han Chinese | Nasal skin | Rhinophyma | No | Yes | Yes |
| S14 | 43 | Man | Asian | Han Chinese | Nasal skin | Rhinophyma | No | Yes | Yes |
| S15 | 39 | Man | Asian | Han Chinese | Nasal skin | Rhinophyma | No | Yes | Yes |

***Notes:***

For hypertrophic scar tissue, five samples of 0.5 cm³ each were taken from the lesion's center. Similarly, five 0.5 cm³ samples were collected from the rhinophyma area in affected patients. For healthy individuals, two 0.25 cm³ samples were taken from normal skin, totaling ten samples.
