# Supplementary material for: Distinct diversity of skin cell populations of rhinophyma and hypertrophic scar illustrated by scRNA-seq
Source: Front Immunol. 2026 Mar 12;17:1703469. doi: 10.3389/fimmu.2026.1703469 (PMC13017343; doi:10.3389/fimmu.2026.1703469)

**Supplementary Figure S1. B cells subgroups in ROS, marker genes, transcriptional traits, and relevant biological processes.**


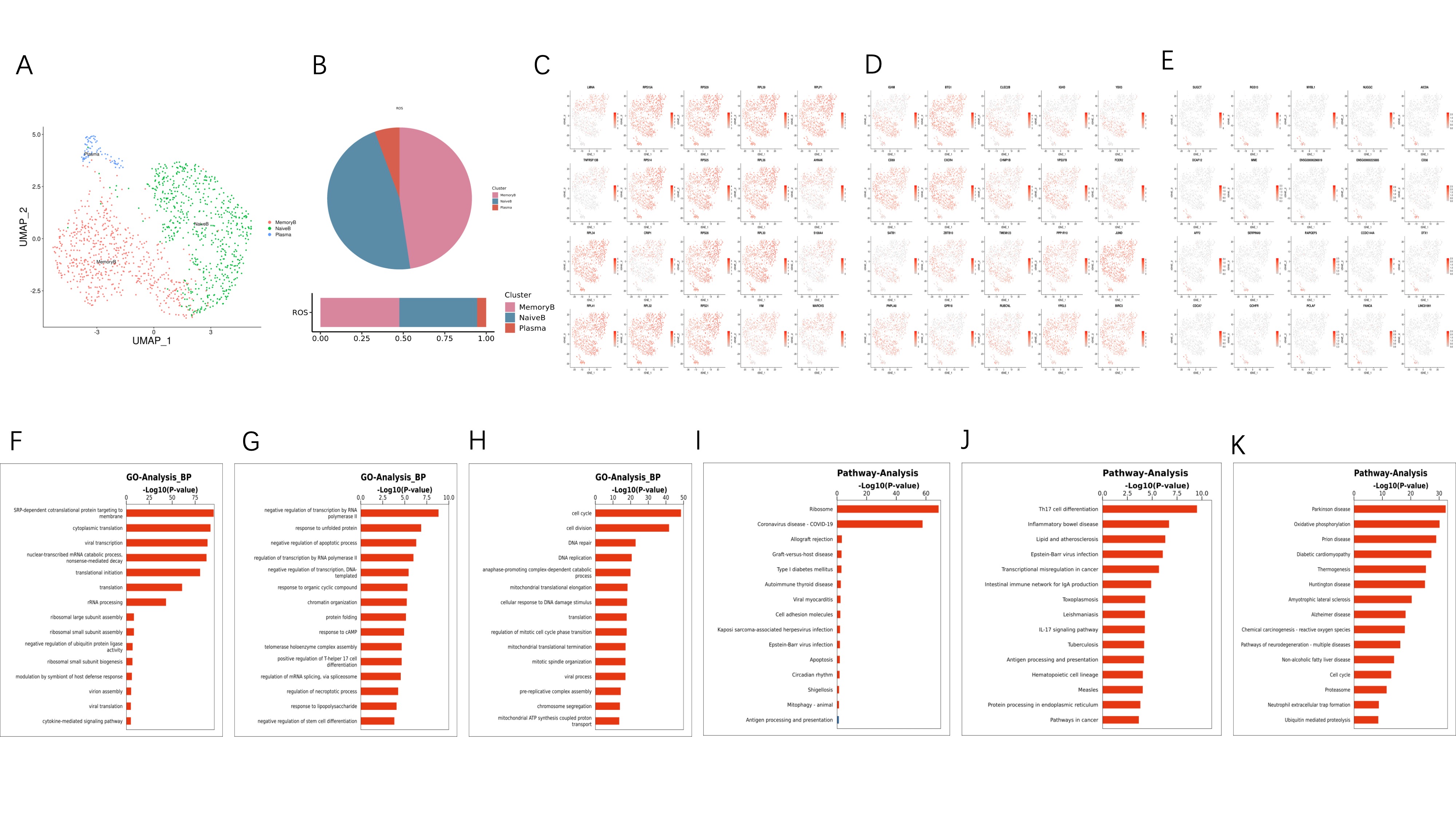

Supplement: Supplementary file 2 [file DataSheet1.docx]
